# REPLANTATION OF THE THUMB OR REVISION OF AMPUTATION: AN EPIDEMIOLOGICAL STUDY

**DOI:** 10.1590/1413-785220243203e274165

**Published:** 2024-08-02

**Authors:** Tiago Guedes da Motta Mattar, Caio Felipe Araujo Matalani, Henrique Brito Silveira, Arthur Kayano Sargaço, Mariana Miranda Nicolosi Pessa, Fernando Vicente de Pontes, Teng Hsiang Wei, Rames Mattar, Marcelo Rosa de Rezende

**Affiliations:** 1 Universidade de São Paulo, Faculdade de Medicina, Instituto de Ortopedia e Traumatologia IOT HCFMUSP, Grupo de Cirurgia da Mão e Microcirurgia Reconstrutiva, São Paulo, SP, Brazil.; 2 Universidade de São Paulo, Faculdade de Medicina, São Paulo, SP, Brazil.; 3 Universidade de São Paulo, Faculdade de Medicina, Instituto de Ortopedia e Traumatologia IOT HCFMUSP, Especialização em Terapia da Mão, São Paulo, SP, Brazil.

**Keywords:** Epidemiology, Replantation, Thumb, Amputation, Microsurgery, Epidemiologia, Reimplante, Polegar, Amputação, Microcirurgia

## Abstract

Objetive: This article presents a retrospective cohort study analyzing patients from IOT-FMUSP who underwent replantation or revision amputation procedures for traumatic thumb amputation between 2013 and 2020. Methods: The study included 40 patients in the replanted group and 41 patients in the amputed group. The patients were divided according to the level of amputation and their medical records were analyzed. Results: A total of 81 patients with digital amputation were analyzed, consisting of 79 males and 2 females, with mean ages of 43 and 49 for the amputed and replanted groups, respectively. According to the Biemer classification, 28.4% had proximal amputation, while 71.6% had distal amputation. The most common occupation was bricklayer (19.75%), and 80.24% were manual workers. Of the patients, 65% returned to their previous work, with 77.77% of them having amputation on their non-dominant hand, mostly caused by circular saw accidents (77.77%). The replantation success rate was 78%, with an average ischemia time of 9 hours and door-to-room time of 2 hours. Conclusion: the study findings revealed that traumatic thumb amputation predominantly affects working-age males with a low education level and the success rate of replantation was high in this ischemia time and door-to-room conditions. ***Level of Evidence II, Retrospective study.***

## RESUMO

Objetivo: Este artigo consiste em um corte retrospectivo que analisou vítimas de amputação traumática do polegar submetidas a reimplante ou procedimentos de regularização da amputação no Instituto de Ortopedia e Traumatologia do Hospital das Clínicas da Faculdade de Medica da Universidade de São Paulo (IOT-FMUSP). Métodos: Foram analisados 40 pacientes reimplantados e 41 pacientes regularizados, que foram separados conforme o nível da amputação e, após, tiveram seus prontuários médicos analisados. Resultados: Foram analisados 81 pacientes com amputação digital (79 homens e 2 mulheres), com idade média de 43 anos e 49 anos (Grupo Amputado e Grupo Reimplante, respectivamente) e 28,4% deles tinham amputação proximal, de acordo com a classificação de Biemer, enquanto 71,6% tinham amputação distal. A ocupação mais comum foi a de pedreiro (19,75%), mas 80,24% eram trabalhadores manuais. 65% dos pacientes retornaram ao trabalho anterior. 77,77% dos pacientes afetaram a mão não dominante, e a serra circular causou 77,77% das amputações. A taxa de sucesso para reimplantes foi de 78%. O tempo de isquemia foi de 9 horas e o tempo de porta-quarto foi de 2 horas. Conclusão: O estudo revelou que as taxas de reimplante foram altas nas condições de isquemia e tempo porta-sala, e a maioria dos pacientes vítimas de amputação traumática do polegar são homens em idade de trabalho e com baixa escolaridade. **Nível de Evidência II, Estudo retrospectivo.**

## INTRODUCTION

 Since the first successful thumb replantation, ^1^ several authors have reported excellent survival rates, ^2^ , ^3^ in addition to a detailed surgical technique. ^4^ The initial objective of finger replantation is to maintain the viability of the finger. ^5^ Another equally important objective is to achieve the most satisfactory function. However, replantation is a complex, expensive procedure that requires a high degree of investment on the part of the patient and society. 

 Amputations of fingers and thumbs are prevalent traumatic injuries affecting North Americans every year. However, replantation rates have gradually dropped in hospitals in the United States of America, due to the challenges of aligning: staff with training in microsurgery, logistics, infrastructure, financial and labor issues. ^6^ , ^7^

 The national literature is scarce in publications of studies on thumb amputation and replantation, with a lack of large series of clinical cases and comparative studies, whether prospective or retrospective. ^8^ Such studies could better illustrate the national reality, and their findings could be confronted with those reported internationally. 

Therefore, we can argue that a descriptive study of thumb replantations and amputations carried out in a large national center with a team trained in Microsurgery, whose patients are part of a different context from other international series of thumb replantations, was necessary.

## OBJECTIVE

The objective of this study was to describe the epidemiological data of patients who were victims of traumatic thumb amputation treated at the Institute of Orthopedics and Traumatology of HC-FMUSP through the procedure of replantation or revision amputation of the amputation stump.

## MATERIALS AND METHODS

The study carried out consists of a retrospective cohort with cross-sectional analysis of patients from the Institute of Orthopedics and Traumatology of FMUSP, victims of traumatic thumb amputation and submitted to replantation or thumb revision amputation procedures, in their different forms and levels, in the period between 2013 and 2020, after the creation and organization of CEMIM (Center for Emergency Care in Reconstructive Microsurgery and Hand Surgery).

The article had the approval of the Committee of Ethics of IOT-FMUSP and it was registered in the Brazil Platform (CAAE: 56255216.4.0000.0068).

### Population studied – Sample Group

After convocation, 81 patients who underwent replantation or amputation of the thumb returned to the outpatient clinic to be evaluated in this research and signed an informed consent form. 40 of these patients underwent thumb replantation (Replanted Group) and 41 underwent thumb amputation with revision of the amputation stump (Amputated Group).

**Table 1.** Sample Groups

Replanted Group	Amputated group
N=40	N=41

All patients who attended the IOT-HC-FMUSP in response to the invitation to participate in the research and who completed all the evaluations included in the research methodology and signed an informed consent form were included.

### Review of medical records and anamnesis

The following parameters were recorded: patient’s age at the time of the surgical procedure, gender, presence of comorbidities, trauma mechanism, time elapsed from trauma to assessment, laterality, ischemia time, type of ischemia, door-to-surgical room time, surgical tactic adopted (including graft use and number of repaired structures), presence of intercurrences and patient habits. Data related to work occupation, manual activities performed before and after surgery and return to work were recorded.

 We adopted the Biemer ^9^ classification to determine the amputation level: Zone 1= nail level; Zone 2 = interphalangeal joint; Zone 3 = proximal phalanx; Zone 4 = Metacarpophalangeal joint; Zone 5 = metacarpus. ^9^

We assessed the handedness of the injured thumb and classified distal injuries by unifying Biemer levels 1, 2 and 3 and proximal injuries by unifying Biemer levels 4 and 5.

## RESULTS

 Of the total number of patients, 79 are male (97.53%) and 2 are female (2.47%). The level distribution of the level of the injury according to the Biemer Classification ^9^ is shown in Table 2 . 

**Table 2.** Distribution of lesion level in patients according to Biemer’s classification.

	I	II	III	IV	V	Total
**Amputated Group**	2	2	20	5	12	41
**Replanted Group**	3	9	22	3	3	40
**Total**	5	11	42	8	15	81

 Regarding the age of the patients, those who underwent thumb revision amputation had a mean age of 43 years-old and those who underwent thumb replantation had a mean age of 49 years-old ( Table 3 ). 

**Table 3.** Distribution of average, minimum and maximum ages of patients.

	Age (average)	Minimum age	Maximum age
**Amputated Group**	43 years-old	16 years-old	73 years-old
**Replanted Group**	49 years-old	21 years-old	71 years-old

 The most common occupation of thumb amputation patients was Bricklayer – Construction Assistant (19.75%). At the time of the accident, out of the total number of patients, 80.24% of the patients were manual workers and 7.4% of the patients were retired. Evaluating Return to Work after the procedure, 28 patients returned to previous work in the Replanted Group (70%) and 25 patients returned to work in the Amputated Group (60.97%) ( Table 4 ). 

**Table 4.** Distribution of patients according to return or absence from work.

	Return to work	Absence from work	Total
**Replanted Group**	28 (70%)	12 (30%)	40
**Amputated Group**	25 (61%)	16 (39%)	41
**Total**	53 (65%)	28 (35%)	81

In terms of education, 53.67% of the patients who underwent thumb revision amputation had incomplete secondary education, and 66.67% of the patients who underwent thumb replantation had incomplete secondary education.

 The distribution found for Trauma Laterality is shown in Table 5 . 

**Table 5.** Distribution of the affected side and patient dominance.

	Left	Right	Total	Dominant	Non-dominant	Total
**Amputated Group**	28	13	41	13	28	41
**Replanted Group**	35	5	40	5	35	40
**Total**	63	18	81	18	63	81

We found 12 patients with comorbidities in the Replanted Group (8 with Systemic Arterial Hypertension, 3 Diabetes Mellitus, 1 Bowel Neoplasm), and 5 in the Amputated Group (5 Systemic Arterial Hypertension + Diabetes Mellitus). Arterial Hypertension and Diabetes mellitus were the most frequent comorbidities (20.98% of patients).

Evaluating the habits of the patients, we found a percentage of 19.75% of smokers, totaling 16 smokers, 10 in the Amputated Group (24.29%) and 6 in the Replanted Group (15%). The number of alcoholics in the sample was 11 (13.58%), 6 in the Amputated Group (14.63%) and 5 in the Replanted Group (12.5%).

Of the total number of Amputated patients (n = 41), 10 patients were previously submitted to the replantation procedure, but without success. When analyzing all of the cases in this sample that were submitted to replantation with success or not (n = 50), compared with the cases of successful replantation (Group Replanted = 40), we obtained a success rate of 80% of the replantations performed.

Out of the complications observed which led to the loss of the replantation, there were 4 cases of venous congestion (9.76%), 5 of arterial ischemia (12.20%), 2 of distal cutaneous necrosis that evolved into non-viability of the thumb (4.88 %) and 1 late necrosis of the thumb (2.44%).

The most common trauma mechanism was amputation by circular saw (n = 63, 77.77%), with 26 cases of Amputated thumbs (63%) and 37 cases of reimplanted thumbs (92.5%). Trauma mechanisms with worse prognosis (explosion, avulsion and crushing) were more common in the Amputated Group (n = 14) (34.14%) than in the Replanted Group (n = 1) (2.5%).

Ischemia time had an average of 8.72 hours in the Amputated Group (MIN = 4, MAX = 19, SD = 3.83, MD = 8) and an average of 8.81 hours in the Replanted Group (MIN = 3, MAX = 16, SD = 3.15).

Door-to-Surgical Room Time had an average of 2.21 hours in the Amputated Group (MIN = 0.5, MAX = 10, SD = 1.5) and an average of 2.18 hours in the Replanted Group (MIN = 1, MAX = 5, SD = 1.2).

The type of ischemia used to preserve the amputated thumb was warm ischemia in 24 cases (30%) and cold ischemia in 56 cases (70%). In the Amputated Group, there were 31 cases of cold ischemia (75.61%) and 10 cases of warm ischemia (24.39%). In the Replanted Group, there were 25 cases of cold ischemia (64.10%) and 14 cases of warm ischemia (35.9%).

Analyzing the surgical technique in the Replanted Group, 2 venous anastomoses were performed in 17 patients (42.5%), 1 venous anastomosis in 9 (22.5%) and in 14 patients there was no data on the number of veins repaired in the surgical description of the procedure (35%). Two arterial anastomoses were performed in 3 patients (7.5%), 1 arterial anastomosis in 31 patients (77.5%) and in 6 patients (15%) there was no data on the number of repaired arteries noted in the medical records. 2 neurorrhaphy procedures were performed in 18 cases (56.25%), 1 neurorrhaphy procedure in 1 case (37.5%) and 0 neurorrhaphy procedures in 2 cases (6.25%). In only 1 case was a vascular graft used and, likewise, in only 1 case was a nerve graft used.

 Most patients were operated on during the Night period (n = 60, 75%). Of the Amputated thumbs, 9 were operated on during the day (21.95%) and 32 at night (78.05%). Of the Replanted thumbs, 11 were operated on during the day (27.5%) and 29 operated on at night (72.5%) ( Table 6 ). 

**Table 6.** Distribution of patients according to the period of care.

	Night duty	Daytime duty	Total
**Replanted Group**	29 (72,5%)	11 (27,5%)	40
**Amputated Group**	32 (78%)	9 (22%)	41
**Total**	61 (75,3%)	20 (24,7%)	81

The average length of hospital stay was 4.68 days in the Amputated Group (MIN = 1, MAX = 21, SD = 5.33), and 6.54 days in the Replanted Group (MIN = 3, MAX = 14, SD = 2, 35).

The average time from the trauma to the functional evaluation in the Amputated Group was 26.27 months (MIN = 12, MAX = 48, SD = 12.08) and in the Replanted Group it was 30.05 months (MIN = 12, MAX = 108, SD = 20.34).

## DISCUSSION

After calling the patients submitted to procedures for replantation and revision amputation of the thumb, 81 patients responded to the request, agreed to participate in this research and signed the free and informed consent form. Out of these patients, 41 had been submitted to revision amputation and 40 to reimplantation. This is the largest series of cases that evaluates, in the same time and service, patients with thumb amputation and replantation.

When evaluating our series, we identified that most of our patients were male (79 of the 81 patients). The higher incidence of traumatic thumb amputation in males is notorious, due to a higher prevalence of this injury in manual workers who use industrial machines (circular saw) which offers an increased risk of these injuries.

Regarding the level of the amputation, we observed a higher relative prevalence of levels 2 and 3 of Biemer in the Replanted Group and levels 4 and 5 in the Amputated Group. We can infer that in lesions at level 2 and 3 (near the interphalangeal region of the thumb) replantation is usually possible, and that probably at level 4 and 5 (metacarpophalangeal region or proximal to it) replantation is less indicated because it is related to crushing or avulsion (mechanisms of greater severity). However, when evaluating these data, it was not possible to demonstrate a statistical difference between amputation levels in the Replanted and Amputated Groups. In our series, level 3 was the most frequent in both the Amputated and Replanted Groups, corresponding to more than half of the cases.

 When comparing the average age of our series with others published in the literature, we observed that our patients are slightly older than most publications (average 43 years-old in the Amputated Group and 49 years-old in the Replanted Group). Glickman et al ^10^ found a mean age of 32.5 years-old and Ciclamini et al ^11^ of 35 years-old. This data is difficult to interpret, however it leads to a reflection on the social conditions of workers in this age group, characteristics of our geographic region (City of São Paulo – Brazil) and of our hospital (university service with high demand for care for patients with high complexity). On the other hand, Chang et al ^12^ found an age group for digital replantations in Taiwan more frequently between 45 and 54 years old. These authors also observed that machines and motorized manual tools caused 68.8% of digital amputations and that higher rates of replantation attempts occurred in thumb amputations (OR: 1.35; p = 0.01), in consultations performed in private hospitals (OR: 1.40; p = 0.01), in specialized medical centers (OR: 2.38; p < 0.001), in regional reference hospitals (OR: 2.41; p < 0.001 ) and in hospitals with annual volume greater than 20 digital amputations (OR: 4.23; p < 0.001). 

 The most common occupation of thumb amputation patients in our series was Bricklayer – Construction Assistant (19.75%). Of the total number of patients, 80.24% of the patients were manual workers at the time of the accident. At the time of the accident, 7.4% of the patients were retired. Other authors ^6 , 7^ draw attention to the incidence of traumatic amputations in the underprivileged population and workers who perform functions related to greater risk. When evaluating the education of our patients, we observed that 53.67% of the patients who underwent revision amputation and 66.67% of the patients who underwent thumb replantation had incomplete secondary education, with no statistically significant difference between the groups, demonstrating a profile like previous literature. 

 Mahmoudi et al ^6^ assess that reimplantation rates have gradually fallen in US hospitals, which makes this procedure less accessible to minorities and the vulnerable population. Unfortunately, we can consider that this is a persistent situation in Brazil, where the opportunity for a patient to undergo a replantation procedure in the public system has always been very low. Mahmoudi et al ^6^ and Hustedt et al ^13^ suggest that the establishment of regional centers for the referral of complex trauma can considerably increase the success of digital replantation in the United States. These authors consider that patients victims of traumatic amputation treated by a surgeon with a high volume of microsurgical procedures in a high-volume center have a 2.5 times greater probability of obtaining a successful replantation. Brazil does not have a regionalized system for caring for patients who are victims of amputations. There is a great lack of high-volume care centers for procedures in reconstructive microsurgery and there is a lack of public policies in this sector. 

 Approximately 30 months after the procedure, 28 patients returned to their previous work in the Replanted Group (70%) and 25 patients returned to work in the Amputated Group (60.97%). Janezic et al ^14^ found a return to the same job as before the thumb replantation procedure in 67% of their patients. Unglaub et al ^15^ reported that most of their 24 treated patients returned to their previous occupation after thumb replantation. Therefore, our results regarding the return to work are like those found by other authors. 

The most common trauma mechanism in our patients was the circular saw (n = 63) (77.77%), with 26 cases of amputated thumbs (63%) and 37 cases of replanted thumbs (92.5%). Trauma mechanisms with worse prognosis (explosion, avulsion and crushing) were more common in the Amputated Group (n = 14) (34.14%) than in the Replanted Group (n = 1) (2.5%). These findings were expected since the mechanism of amputation by circular saw is usually related to a higher rate of indication and success for the replantation procedure.

When assessing handedness, 13 patients who underwent revision amputation (31.72%) and 5 who underwent replantation (12.5%) had the dominant side affected. We did not observe studies related to the side and dominance in the literature, but when analyzing our data, we can infer that there is a slightly more frequent occurrence of amputation of the dominant side in cases of revision amputation. Considering that the circular saw was the most frequent mechanism of amputation, mainly in the Replanted Group (92.5%), it is usually the non-dominant side that approaches the machine in a more dangerous way (holding the object to be cut) being therefore the most vulnerable side. The worst prognosis mechanisms (avulsion and crushing), more frequent in the Amputated Group, occur more frequently in the dominant limb. Despite the differences pointed out, it was not possible to demonstrate statistical differences between the groups.

 Of all patients, 12 had comorbidities in the Replanted Group (SAH: 8 and DM: 3) and 5 had comorbidities in the Amputated Group (SAH and DM: 5). Hypertension (SAH) and Diabetes Mellitus (DM) were the most frequent comorbidities. Hustedt et al ^13^ consider that patients with more than 3 comorbidities and those with a history of alcohol abuse, anemia, electrolyte imbalance, obesity, peripheral vascular disease, or psychotic disorders, are at greater risk of replantation failure and post-operative complications. 

Assessing the patients’ habits, we found 16 smokers (19.75% of smokers), 10 in the Amputated Group (24.29%) and 6 in the Replanted Group (15%). The number of alcoholics was 11 (13.58%), 6 in the Amputated Group (14.63%) and 5 in the Replanted Group (12.5%). Despite the higher prevalence of smokers and alcoholics in the Amputated Group, it was not possible to demonstrate statistical difference between the groups in relation to these variables.

 Of the total number of amputated patients (n = 41), 10 were previously submitted to the replantation procedures, but without success in terms of viability. In this series, of the 51 patients who underwent the replantation procedures, 40 were successful. Therefore, in this series we obtained a success rate of 78.43% for thumb replantations performed during the study period. We found a wide variation in the success rate of thumb replantations noted in the medical literature. Schlenker et al ^2^ reveal a survival rate of 73%, Arakaki and Tsaial ^16^ 71%, Zumiotti et al ^17^ 68.75%, Mattar Jr et alt ^8^ of 64 % of replantations in amputations caused by avulsion mechanism, Zumiotti et al ^17^ of 73% of replantations in the topography of the distal phalanx of the thumb, Janezic et al ^14^ of 66%, Sharma et al ^3^ of 91.30%, Agarwal et al ^18^ 92%, Mahmoudi et al ^6^ 74% between 2004 and 2006, and 65% between 2010-2012. When comparing our data with those in the literature, considering that our service is university-based, with high demand, where replantations are more frequently indicated, our survival rate can be considered like that of most publications. 

 Out of the complications observed, which led to the loss of the reimplantation, there were 4 cases of venous congestion (9.76%), 5 of arterial ischemia (12.20%), 2 of distal cutaneous necrosis that made the reimplantation unfeasible (4.88%). and 1 late necrosis of the thumb (2.44%). Arakaki and Tsai ^16^ reported the need for re-exploration in 16.3% of the reimplanted thumbs due to vascular impairment, and nine of these were recovered (45%). Sharma et al ^3^ emphasize that early re-exploration of vascular problems generates a high recovery rate, and should be performed in all cases, and recommend the use of vein grafts in more severe injuries due to crushing and avulsion. In our sample, the number of arterial and venous vascular complications was similar. Although we constitute a hospital with a high volume of microsurgical procedures, in our series there are no cases of success in re-exploration due to vascular complications. We consider that we have difficulties in carrying out re-explorations in our public service, which faces infrastructure problems. 

Ischemia time had an average of 8.72 hours in the Amputated Group (MIN = 4, MAX = 19, SD = 3.83) and an average of 8.81 hours in the Reimplanted Group (MIN = 3, MAX = 16, SD = 3.15). There is no difference between the groups, and this time can be considered adequate for an urban center of a metropolis like São Paulo. However, it is still a long time by international standards, revealing a lack of infrastructure in the care of these patients.

In our series, we found a door-to-surgical room time with an average of 2.21 hours in the Amputated Group (MIN = 0.5, MAX = 10, SD = 1.5) and an average of 2.18 hours in the Replanted Group (MIN = 1, MAX = 5, SD = 1,2). Therefore, we did not observe differences between the groups regarding the waiting time between the patient’s arrival at the hospital and the arrival in the operating room. This time of approximately 2 hours and 20 minutes could certainly be reduced with the improvement of the infrastructure in our hospital.

Evaluating our patients, we observed that the ischemia of the amputated part until arrival at the hospital was warm in 24 cases (30%). This data also clearly reveals the lack of adequate infrastructure to the care for this type of trauma in our country.

 We can state that the surgical techniques and tactics for treating thumb amputations in our group are standardized and may vary in the indication criteria for the use of vascular grafts and peripheral nerve repair techniques. In most cases, the repair of vascular, nervous, tendinous and osteoarticular system structures depends on the anatomy of the injury and the trauma mechanism. In the group of replanted patients, 2 venous anastomoses were performed in 17 patients (42.5%), 1 venous anastomosis in 9 (22.5%) and in 14 patients (35%) we did not find data in the medical records on the number of veins repaired. Efanov et al ^19^ draw attention to the fact that all efforts should be made to favor the repair of two veins, since, in this condition, they find a better survival of replanted fingers. The approach adopted in our group is to repair as many vascular structures as possible, whether arterial or venous. In our patients, 2 arterial anastomoses were performed in 3 patients (7.5%), 1 arterial anastomosis in 31 patients (77.5%) and in 6 patients (15%) we did not find data in the medical records about the number of repaired arteries. 

 In the same way as Schlenker et al, ^2^ we used a vein graft connecting the radial artery (proximally) to the thumb arteries (distally) to circumvent a traumatized area in 1 patient. In injuries that do not involve an avulsion mechanism, this procedure may be unnecessary, being bone shortening for bone revision amputation usually sufficient to allow vascular and nerve anastomosis with no tension in preserved and viable structures. This tactic was used in most patients in this study. However, we agree with Barbato and Salsak ^4^ on the need to follow a flow of procedures depending on the type of traumatic thumb amputation, including the use of anastomosed vascular graft in the amputated segment as the initial procedure in more complex traumatic amputations (especially in avulsion injuries). 

In our series, 2 neurorrhaphy procedures were performed in 18 cases (56.25%), 1 neurorrhaphy procedure in 1 case (37.5%) and 0 neurorrhaphy procedures in 2 cases (6.25%). In only 1 case, a vascular graft was used, and in 1 case, a nerve graft was used in the acute phase of reimplantation.

Most of our cases were operated during the Night duty period (n = 61) (75.3%). We can infer that most patients suffer the accident during the day and that, after just over 8 hours of ischemia, they are submitted to the replantation or revision amputation procedure at night. Of the amputated thumbs, 9 were operated on during the day (21.95%) and 32 at night (78.05%). Of the replanted thumbs, 11 were operated on during the day (28.21%) and 28 operated on at night (71.79%), with no statistically significant difference between the groups.

Regarding patient satisfaction with the procedure performed, 32 patients declared themselves satisfied with the revision amputation procedure (72%) and 38 satisfied with the replantation (95%). When comparing the satisfaction index of our patients who underwent thumb replantation with those presented in other publications, we again found similar values.

## CONCLUSIONS

The epidemiological data evaluated in this study revealed that most patients who are victims of traumatic thumb amputation are males in working age and with low education level. About 20% of patients have comorbidities. The most frequent amputation level was at the proximal phalanx. More proximal amputations were more frequent in the amputated group. The circular saw was the most frequent mechanism, and the most common occupation was Bricklayer-Construction Assistant. Most amputations occurred in the non-dominant thumb. Ischemia time was approximately 9 hours and door-to-room time 2 hours. The type of ischemia was cold in 70% of patients and hot in 30%. Most replantation and revision amputation procedures occurred during the night duty period (75%). The success rate for replantations was 78%. The most frequent complications were arterial and venous thrombosis (20%). Hospitalization time was approximately 6 days in the replanted group and 4 days in the amputated group. The satisfaction rate was higher in the replanted group (95%) than in the amputated group (78%). Most patients returned to their previous work: 70% in the replanted group and 60% in the amputated group.

## References

[1] Komatsu S., Tamai S. (1968). Successful replantation of a completely cut-off thumb. Plastic and Reconstructive Surgery.

[2] Schlenker J. D., Kleinert H. E., Tsai T. M. (1980). Methods and results of replantation following traumatic amputation of the thumb in sixty-four patients. J Hand Surg Am.

[3] Sharma S., Lin S., Panozzo A., Tepper R., Friedman D. (2005). Thumb replantation: a retrospective review of 103 cases. Ann Plast Surg.

[4] Barbato B., Salsac A. V. (2020). Finger and thumb replantation: from biomechanics to practical surgical applications. Hand Surg Rehabil.

[5] Morrison W. A., O’Brien B. M., MacLeod A. M. (1978). Digital replantation and revascularisation. A long term review of one hundred cases. Hand.

[6] Mahmoudi E., Swiatek P. R., Chung K. C., Ayanian J. Z. (2016). Racial variation in treatment of traumatic finger/thumb amputation: a national comparative study of replantation and revision amputation. Plast Reconstr Surg.

[7] Mahmoudi E., Huetteman H. E., Chung K. C. (2017). A population-based study of replantation after traumatic thumb amputation, 2007-2012. J Hand Surg Am.

[8] Mattar R., Azze R. J., Paula E. J. L., Kimura L. K., Okane S. Y., Rezende M. R., Starck R., Canedo A. C. (1995). Reimplantes de polegar nas amputações provocadas por mecanismo de avulsão. Revista Brasileira de Ortopedia.

[9] Biemer E. (1980). Definitions and classifications in replantation surgery. Br J Plast Surg.

[10] Glickman L. T., Mackinnon S. E. (1990). Sensory recovery following digital replantation. Microsurgery.

[11] Ciclamini D., Tos P., Magistroni E., Panero B., Titolo P., Da Rold I. (2013). Functional and subjective results of 20 thumb replantations. Injury.

[12] Chang D. H., Ye S. Y., Chien L. C., Ma H. (2015). Epidemiology of digital amputation and replantation in Taiwan: a population-based study. J Chin Med Assoc.

[13] Hustedt J. W., Bohl D. D., Champagne L. (2016). The detrimental effect of decentralization in digital replantation in the united states: 15 years of evidence from the national inpatient sample. J Hand Surg Am.

[14] Janezic T. F., Arnez Z. M., Solinc M., Zaletel-Kragelj L. (1996). One hundred sixty-seven thumb replantations and revascularisations: early microvascular results. Microsurgery.

[15] Unglaub F., Demir E., Von Reim R., Van Schoonhoven J., Hahn P. (2006). Long-term functional and subjective results of thumb replantation. Microsurgery.

[16] Arakaki A., Tsai T. M. (1993). Thumb replantation: survival factors and re-exploration in 122 cases. J Hand Surg Br.

[17] Zumiotti A. V., Ohno P. E., Guarnieri M. V., Prada F. (1995). Reimplante de dedos em amputações distais à articulação interfalângica distal. Rev Bras Ortop.

[18] Agarwal J. P., Trovato M. J., Agarwal S., Hopkins P. N., Brooks D., Buncke G. (2010). Selected outcomes of thumb replantation after isolated thumb amputation injury. J Hand Surg Am.

[19] Efanov J. I., Rizis D., Landes G., Bou-Merhi J., Harris P. G., Danino M. A. (2016). Impact of the number of veins repaired in short-term digital replantation survival rate. J Plast Reconstr Aesthet Surg.

